# Clinical Performance of High-Impact 3D-Printed Denture Resins: A Multicenter Cohort With Survival Analysis

**DOI:** 10.7759/cureus.113768

**Published:** 2026-08-01

**Authors:** Eric D Kukucka, Sundeep Rawal, Nassif Youssef, Arwinder Judge, Joshua Dartez, Gregori M Kurtzman

**Affiliations:** 1 Prosthodontics, Aspen Dental, Chicago, USA; 2 Prosthodontics, Aspen Dental, Merritt Island, USA; 3 Prosthodontics, Aspen Dental, Oswego, USA; 4 General Dentistry and Implantology, Private Practice, Silver Spring, USA

**Keywords:** 3d-printed dentures, clinical outcomes, denture base resins, denture fracture, prosthesis design, removable prosthodontics, workflow variability

## Abstract

Background

The clinical performance of 3D-printed denture base resins remains incompletely defined under real-world conditions, where workflow variability may influence outcomes beyond material properties alone.

Objective

This study aimed to compare the clinical performance of Lucitone Digital Print (LDP) (Dentsply Sirona, Charlotte, NC, USA) and SprintRay High Impact (SRHI) (SprintRay, Los Angeles, CA, USA) 3D-printed dentures in a multicenter cohort. The primary objective was to evaluate functional retention in vivo. Secondary objectives included comparison of fracture-free survival, fracture incidence, time to fracture, failure etiology, and assessment of workflow-related factors that may influence clinical outcomes independent of material properties.

Methods

A cohort of 1,011 dentures (510 LDP, 501 SRHI) was followed through December 31, 2025. Functional retention in vivo was the primary endpoint. Kaplan-Meier survival analysis, log-rank testing, and Cox proportional hazards modeling were performed. Secondary outcomes included fracture-free survival, cumulative breakage rates, time to fracture, and root cause analysis.

Results

At the final follow-up, 68.0% of LDP and 52.3% of SRHI units remained in vivo. SRHI demonstrated significantly greater functional discontinuation (HR: 1.79; 95% CI: 1.47-2.18; P<.001). Fracture rates were similar between groups (13.3% vs 12.6%), and fracture-free survival did not differ significantly (P=.340). Failures were predominantly associated with prosthesis design and workflow variables.

Conclusion

Clinical outcomes of 3D-printed dentures are driven more by workflow execution and prosthesis design than by material selection alone.

## Introduction

Digital prosthodontics has transitioned removable prosthetic fabrication from an analog process dependent on physical impressions, stone casts, flasking, and heat-polymerized polymethyl methacrylate (PMMA) to digital workflows based on intraoral scanning, computer-aided design (CAD), and additive manufacturing. These workflows offer advantages in production efficiency, reproducibility, archiving, and rapid remanufacture. However, the clinical acceptance of 3D-printed dentures depends not only on workflow convenience but also on their ability to withstand functional loading in vivo [1,2].

Conventional PMMA remains the historical reference standard because of its long clinical history, but contemporary printed denture resins have been modified to improve flexural behavior, impact resistance, and fracture toughness [3-9].

The literature has documented that 3D-printed denture base polymers may demonstrate mechanical properties that differ from conventional and milled materials, with performance influenced by resin formulation, print orientation, layer thickness, post-polymerization, and aging [10-14].

## Materials and methods

**Figure 1:**
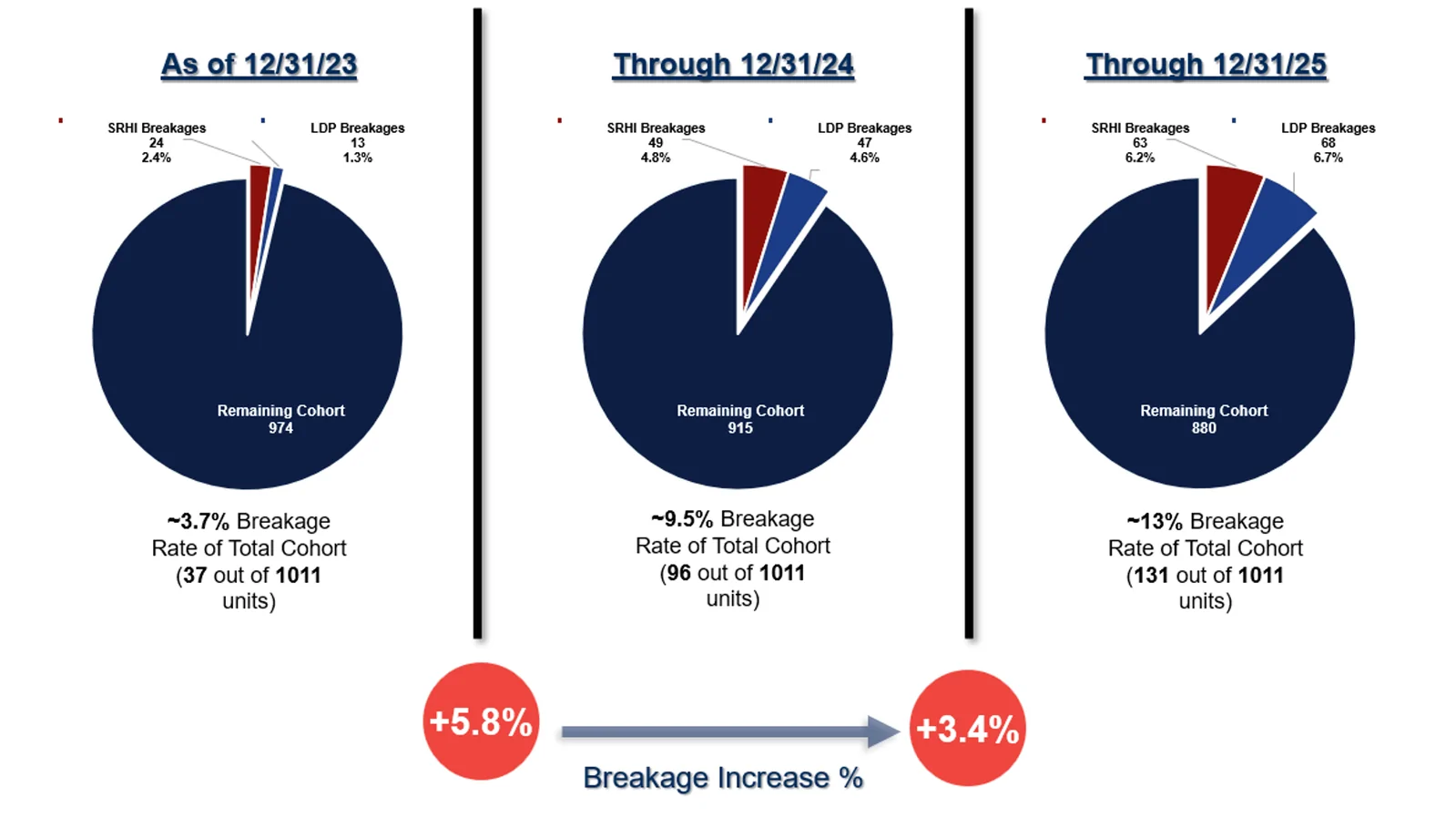
Material gate tracking through 2025 revealed a 3.4% breakage rate increase from 2024. Breakage rate=cumulative broken arches÷total cohort arches (1,011 arches: LDP (n=510) and SRHI (n=501)). Cumulative broken arches to date: 131 (LDP 68, SRHI 63). Time-to-failure mean±SD: 414.6±283.9 days. LDP: Lucitone Digital Print; SRHI: SprintRay High Impact

The present material gate cohort was designed to compare the long-term clinical performance of Lucitone Digital Print (LDP) (Dentsply Sirona, Charlotte, NC, USA) and SprintRay High Impact (SRHI) (SprintRay, Los Angeles, CA, USA) dentures in a multicenter clinical environment. The primary objective was to compare functional retention in vivo between the two materials. Secondary objectives included evaluation of fracture-free survival, fracture incidence, time to fracture, root cause analysis, and the influence of digital workflow variables, fabrication pathway, and prosthesis indication on clinical outcomes. Figure 1 illustrates functional retention for the full cohort and provides the primary time-to-event visualization used to interpret long-term clinical service.

Digital workflow variability as a primary determinant of clinical outcome

In contrast to conventional prosthodontic fabrication, additive denture workflows introduce multiple operator-dependent and system-dependent variables that may influence clinical performance. Digital workflows can be considered as four sequential stages: data acquisition, virtual design, manufacturing, and post-processing. Each stage may introduce small deviations that can propagate through the workflow [15-19].

Intraoral scanning accuracy depends on operator technique, soft tissue control, saliva management, and the ability to capture the full vestibular extension and functional sulcus without distortion. Errors at this stage may result in compromised intaglio adaptation and uneven tissue support [20-25]. CAD parameters, including denture base thickness, palatal contour, flange form, tooth position, and occlusal scheme, must be deliberately evaluated because digital design does not provide the same tactile reinforcement cues available during conventional wax-up procedures [21-24]. Manufacturing variables, including print orientation, layer thickness, support placement, and build-platform calibration, may alter internal stress distribution and interlayer bonding [15-19]. Finally, washing, drying, post-curing time, light intensity, and temperature may affect polymer conversion and the final mechanical properties of the denture base [3-7].

Methodology

This multicenter cohort included 1,011 printed denture units delivered during the material gate evaluation. The LDP cohort included 510 units, while the SRHI cohort included 501 units. LDP dentures were produced through centralized laboratory workflows, whereas SRHI dentures included centralized and in-office workflows, reflecting routine clinical adoption patterns. Denture types included definitive and immediate prostheses, with subsequent analyses recognizing that immediate dentures may be removed for planned delivery of a definitive prosthesis rather than for mechanical failure. The SRHI cohort included a higher proportion of immediate dentures and in-office workflows, which may have influenced early discontinuation independent of material performance.

The primary endpoint was functional retention in vivo, defined as continued service at the last follow-up. Units recorded as no longer in vivo were treated as events for the primary retention analysis, while units remaining in vivo on December 31, 2025, were censored at the last follow-up. A secondary fracture-free sensitivity analysis treated denture fracture as the event of interest and treated nonfracture discontinuation as censoring. Time was measured in days from delivery to the endpoint or censoring. Kaplan-Meier survival curves were generated for both endpoints [20]. Between-cohort comparisons were performed with log-rank testing. Cox proportional hazards modeling was used to estimate univariable hazard ratios, with LDP as the reference group. Multivariable adjustment was not performed because complete covariate-level data were not available across all sites. This included removal for fracture, remake, or planned transition to definitive prostheses.

**Figure 2:**
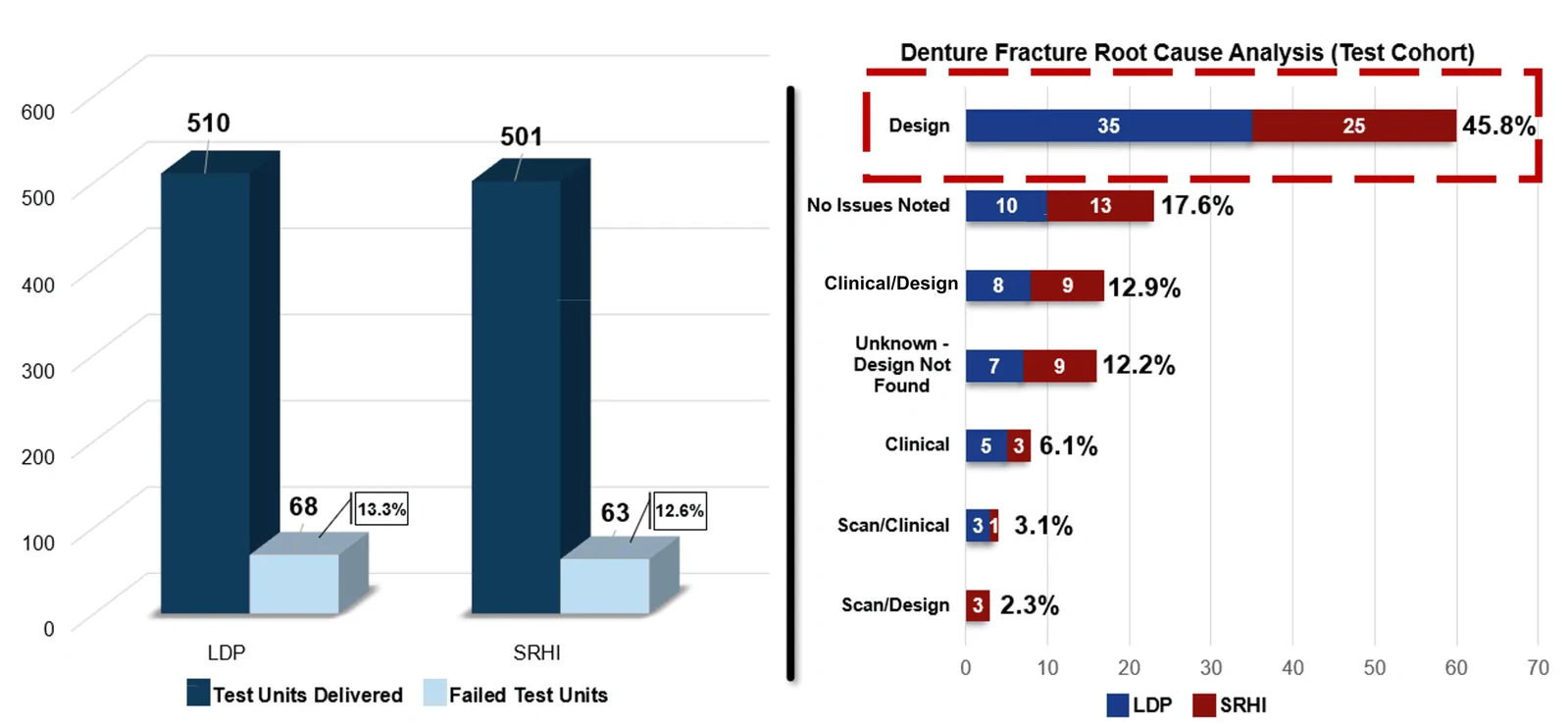
Overall breakage rates remained low, with design quality remaining the main contributing factor to fractures. Total broken arches: n=131 (LDP n=68/510 (13.3%); SRHI n=63/501 (12.6%)). Time-to-failure mean±SD: LDP 456.9±292.8 (n=68); SRHI 368.9±266.6 (n=63); combined 414.6±283.9 days. LDP: Lucitone Digital Print; SRHI: SprintRay High Impact

**Figure 3:**
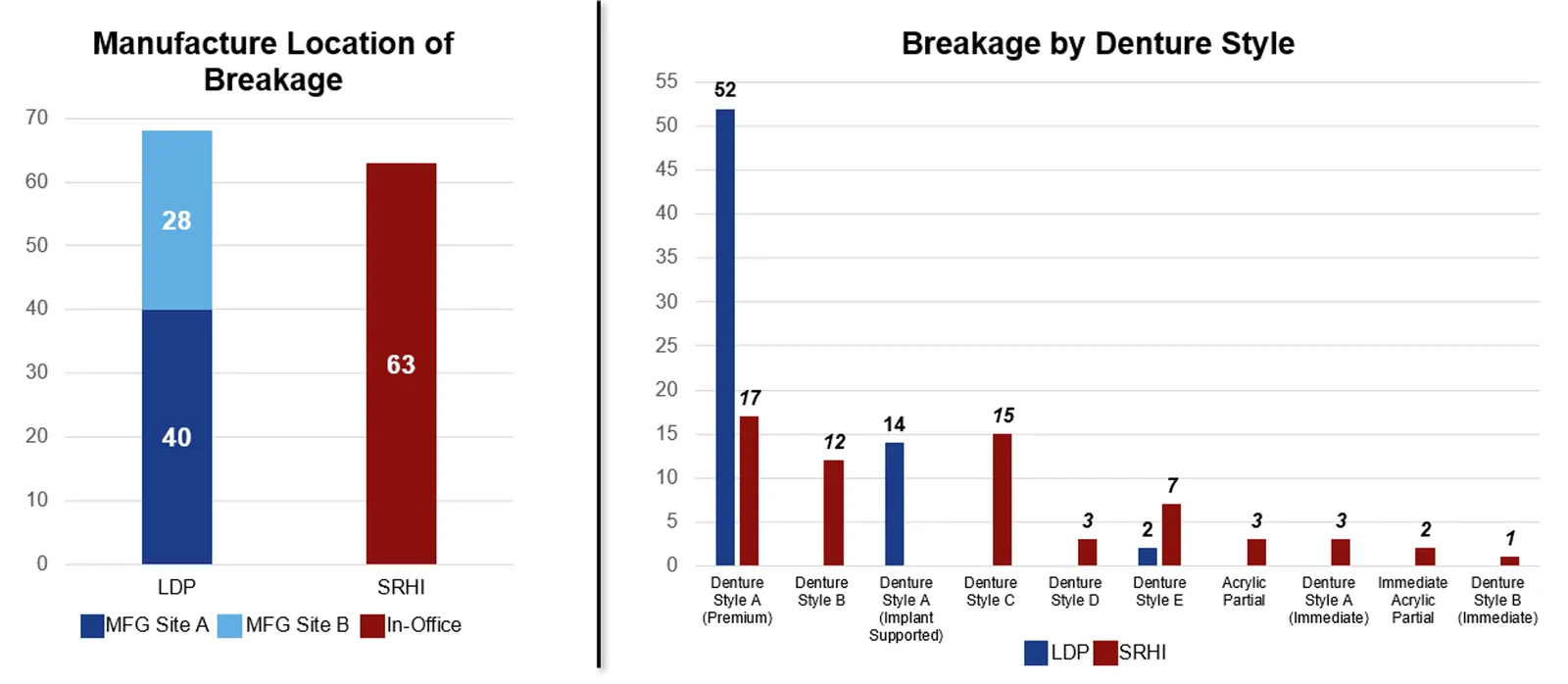
Cohort denture breakage analysis. LDP n=510 arches; SRHI n=501 arches; total broken arches: n=131 (LDP 68, SRHI 63). Time-to-failure mean±SD: LDP 456.9±292.8 (n=68); SRHI 368.9±266.6 (n=63); combined 414.6±283.9 days. LDP: Lucitone Digital Print; SRHI: SprintRay High Impact; MFG: manufacturing

Descriptive analyses included cumulative breakage rate, mean and median time to fracture, root cause distribution, and status at the last follow-up. Root cause categories included design, clinical, clinical/design, scan/clinical, scan/design, unknown or design not found, and no issues noted. Figure 2 illustrates fracture-free survival, Figure 3 illustrates cumulative breakage through 2025, Figure 4 summarizes root cause categories, and Figure 5 displays time-to-fracture distribution among fractured units. Inter-examiner calibration was not formally assessed.

**Figure 4:**
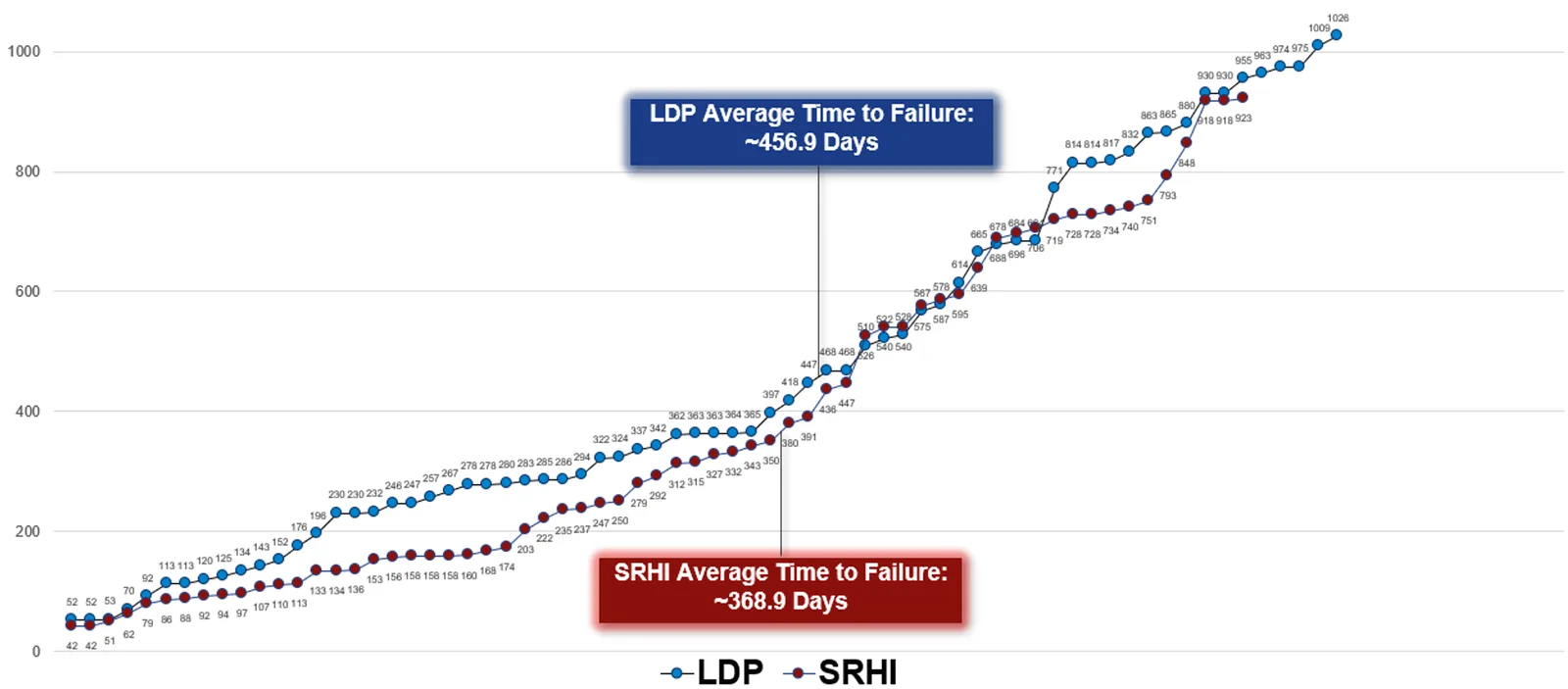
Time to failure: cohort denture breakages. Time to failure=days from delivery to end of service. Time-to-failure mean±SD: LDP 456.9±292.8 (n=68); SRHI 368.9±266.6 (n=63); combined 414.6±283.9 days. LDP: Lucitone Digital Print; SRHI: SprintRay High Impact

**Figure 5:**
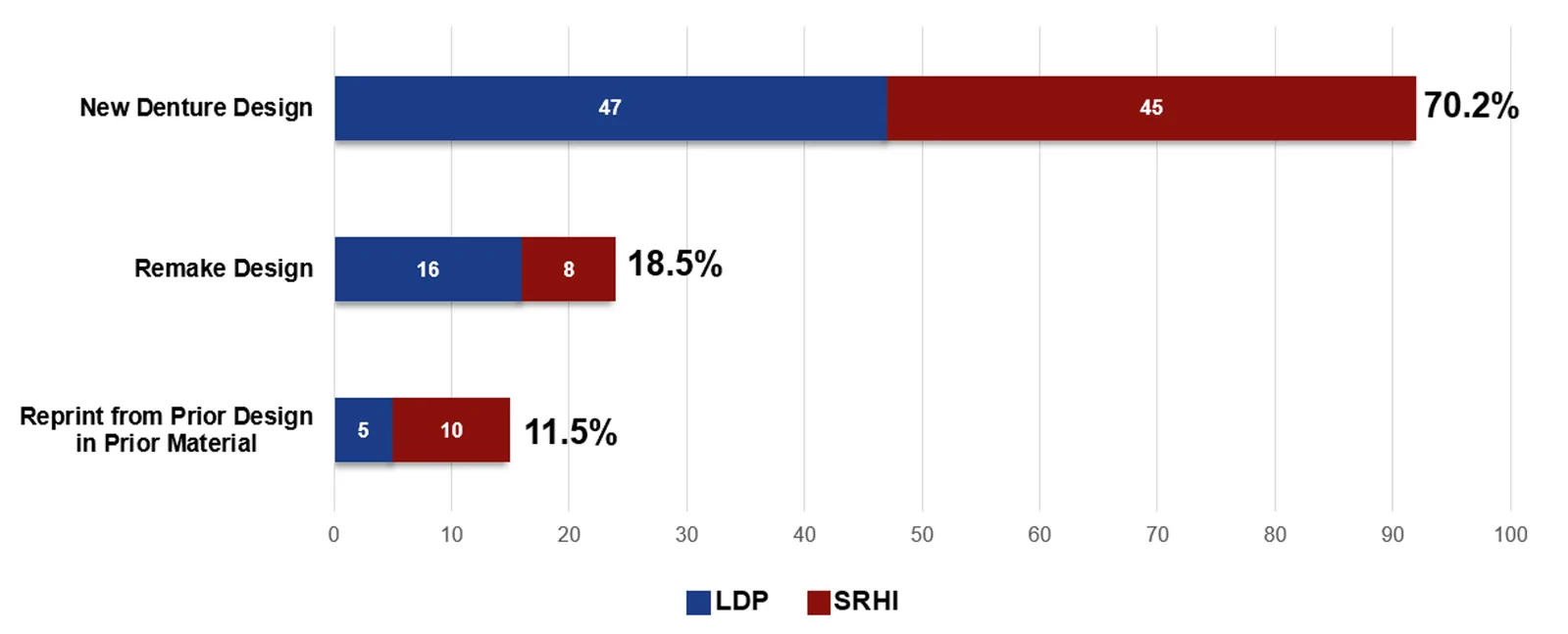
Cohort group: denture type prior to breakage. Denture style at fabrication tabulated across 131 broken arches (LDP n=68; SRHI n=63). Combined time to failure: 414.6±283.9 days (range 42-1026). LDP: Lucitone Digital Print; SRHI: SprintRay High Impact

Data collection and outcome assessment

Clinical data were collected through the material gate tracking system and participating clinical sites using standardized outcome definitions. Functional status, fracture events, and reasons for discontinuation were documented throughout the observation period. Root cause analysis was performed using predefined categories that included design-related, clinical, scan-related, combined clinical/design, combined scan/design, unknown/design not identified, and no issues identified. Cases with incomplete information were retained in survival analyses when outcome status was known; observations without an endpoint at study completion were censored at the last documented follow-up.

All data were collected and measured using Microsoft Excel (Microsoft Corp., Redmond, WA, USA); charts and graphs were created using Microsoft PowerPoint (Microsoft Corp., Redmond, WA, USA).

## Results

The full cohort consisted of 1,011 dentures, of which 510 were fabricated using LDP and 501 using SRHI. At the final follow-up, 347 LDP units (68.0%) and 262 SRHI units (52.3%) remained in vivo, while functional discontinuation was observed in 163 LDP units and 239 SRHI units (Table 1). Kaplan-Meier functional retention analysis demonstrated earlier and greater discontinuation in the SRHI cohort, with a statistically significant difference identified by log-rank testing (χ²=33.53; P<.001) (Figure 1). Univariable Cox proportional hazards modeling further demonstrated that SRHI was associated with a significantly higher hazard of discontinuation compared with LDP (hazard ratio (HR): 1.79; 95% confidence interval (CI): 1.47-2.18; P<.001) (Table 2).

**Table 1:** Cohort status and outcome summary through December 31, 2025. LDP: Lucitone Digital Print; SRHI: SprintRay High Impact

Outcome	LDP	SRHI	Overall
Units delivered	510	501	1011
Units remaining in vivo	347 (68.0%)	262 (52.3%)	609 (60.2%)
Units no longer in vivo	163	239	402
Denture fractures	68 (13.3%)	63 (12.6%)	131 (13.0%)
Mean time to fracture, days	456.9	368.9	414.6
Median time to fracture, days	363	292	332

**Table 2:** Survival analyses. LDP served as the reference group in Cox proportional hazards models. LDP: Lucitone Digital Print; SRHI: SprintRay High Impact; HR: hazard ratio; CI: confidence interval

Endpoint	Event definition	HR for SRHI vs LDP	95% CI	Log-rank chi-square	P-value
Functional retention	No longer in vivo	1.79	1.47-2.18	33.53	< .001
Fracture-free survival	Denture fracture	1.18	0.84-1.67	0.91	.340

When fracture alone was considered as the endpoint, survival curves showed substantially reduced separation between cohorts, indicating that discontinuation pathways rather than structural failure accounted for the majority of differences observed in functional retention. In fracture-free sensitivity analysis, SRHI demonstrated only a modestly higher fracture hazard compared with LDP (Table 2) (HR: 1.18; 95% CI: 0.84-1.67; P=.341), and the log-rank comparison did not reach statistical significance (χ²=0.91; P=.340) (Figure 2). These findings indicate that the observed difference in functional retention was influenced substantially by prosthesis indication, planned transition to definitive prostheses, and workflow-related factors, particularly within immediate denture protocols.

Fracture occurred in 68 LDP dentures (13.3%) and 63 SRHI dentures (12.6%) (Table 1). The cumulative breakage rate increased progressively from approximately 3.7% in 2023 to 9.5% in 2024 and 13.0% in 2025, demonstrating a gradual but controlled increase in fracture incidence over time (Figure 3). Root cause analysis demonstrated that design quality was the most frequently identified contributor to fracture, exceeding purely clinical or scan-related causes (Figure 4). Design-related categories included inadequate denture base thickness, insufficient reinforcement of high-stress regions, occlusal scheme deficiencies, and combined clinical/design factors. The distribution of root causes was not isolated to a single material, supporting the interpretation that workflow execution and prosthesis design were primary determinants of failure rather than resin composition alone. Time-to-fracture analysis demonstrated longer mean and median survival for LDP compared with SRHI (Table 1), with mean times of 456.9 days and 368.9 days and median times of 363 days and 292 days, respectively (Figure 5). The broader distribution of later-occurring failures in the LDP cohort suggests improved long-term mechanical stability once the initial adaptation phase was surpassed. The observed fracture rate is consistent with reported ranges for conventional denture base materials.

**Figure 6:**
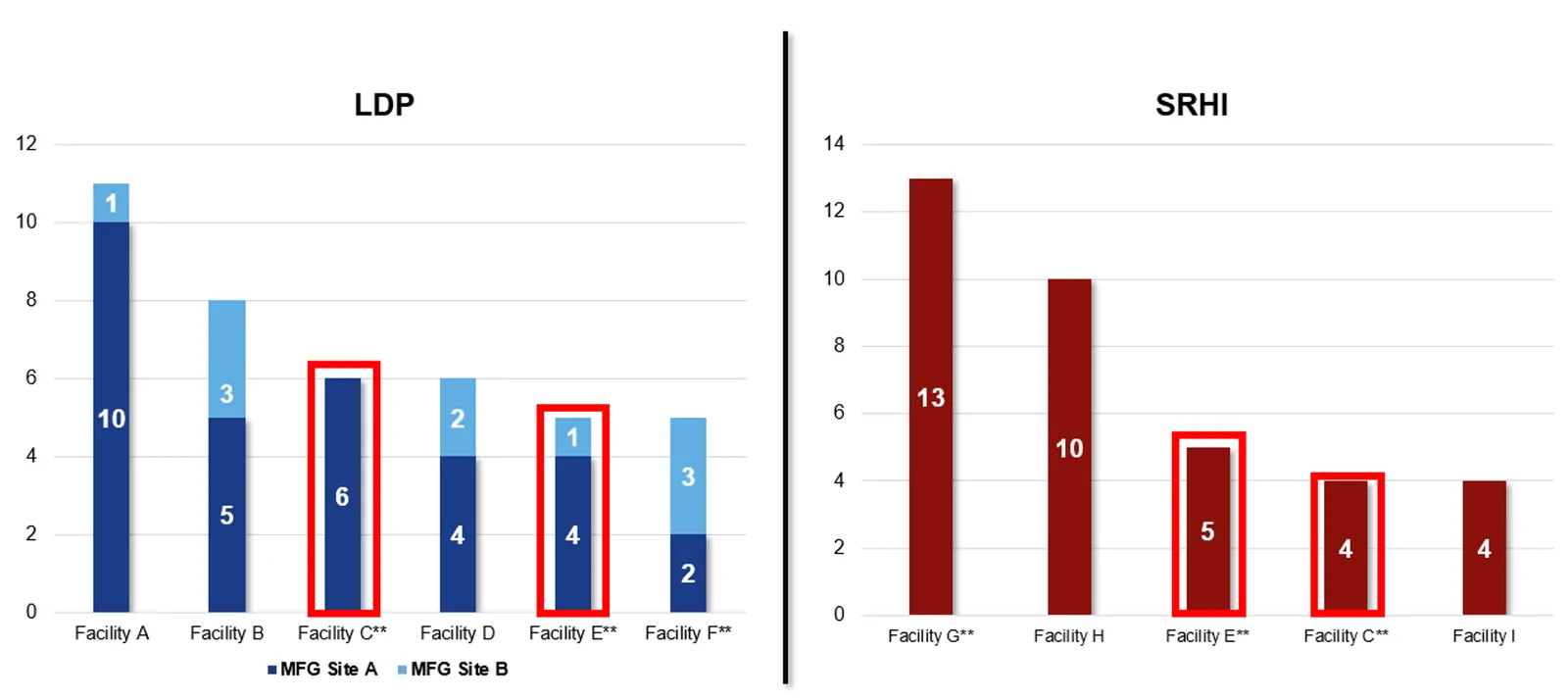
Cohort denture breakages: LDP versus SRHI. **Offices with in-office designers at fabrication. Offices ranked by broken-arch count; total broken arches: n=131 (LDP 68, SRHI 63). Time-to-failure mean±SD: LDP 456.9±292.8 (n=68); SRHI 368.9±266.6 (n=63); combined 414.6±283.9 days. LDP: Lucitone Digital Print; SRHI: SprintRay High Impact; MFG: manufacturing

**Figure 7:**
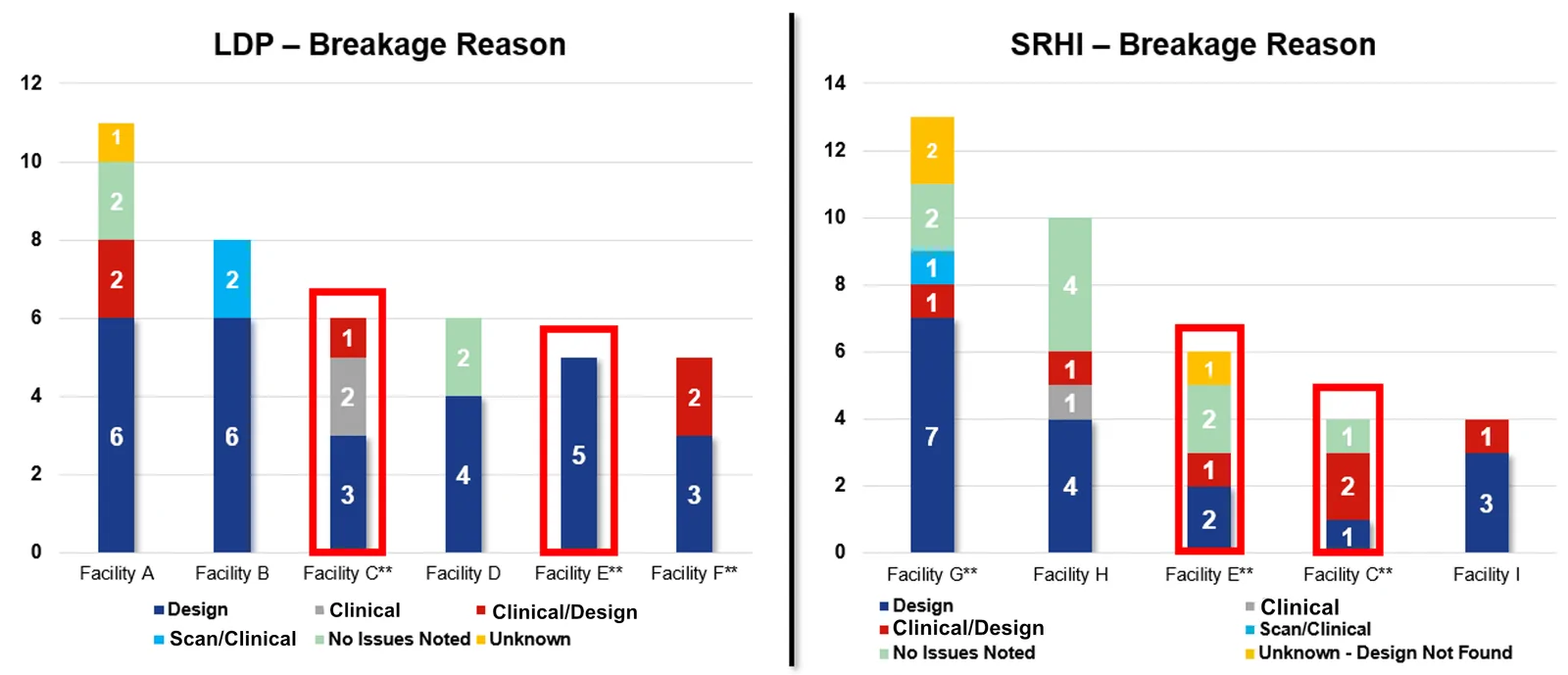
Cohort denture breakage reason: LDP versus SRHI. **Offices with in-office designers at fabrication. Offices ranked by broken-arch count; total broken arches: n=131 (LDP 68, SRHI 63). Time-to-failure mean±SD: LDP 456.9±292.8 (n=68); SRHI 368.9±266.6 (n=63); combined 414.6±283.9 days. LDP: Lucitone Digital Print; SRHI: SprintRay High Impact

**Figure 8:**
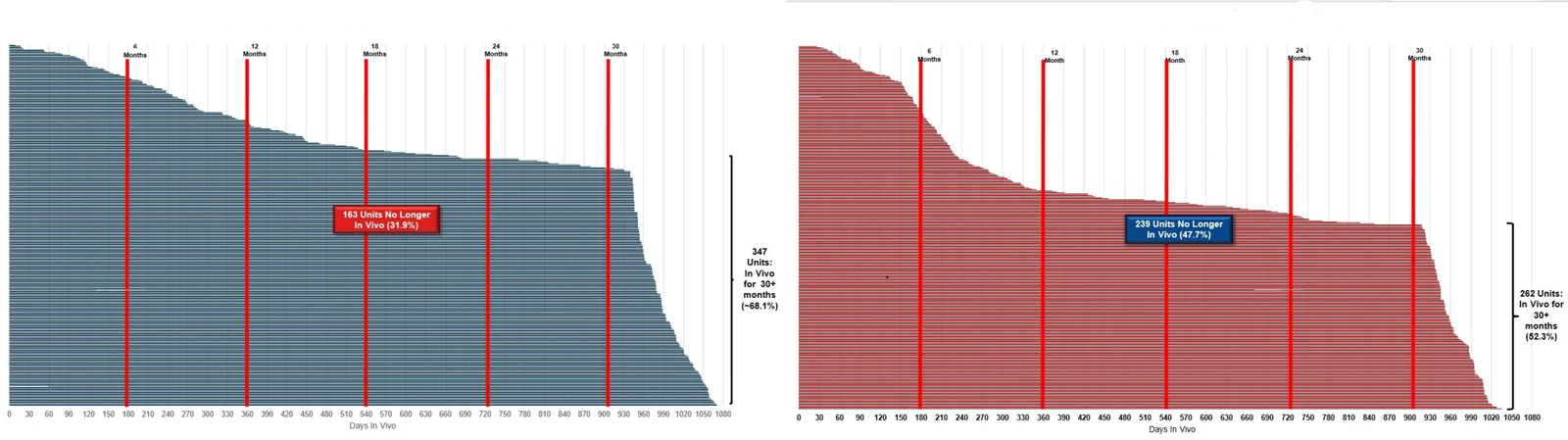
LDP material gate cohort (left) and SRHI material gate cohort (right) time in vivo. LDP cohort: n=510 arches; in vivo status as of 12/31/2025; 30-month threshold=material gate long-term survival benchmark. Days in vivo (all arches): 762±338 (range 9-1072) (left). SRHI cohort: n=501 arches; in vivo status as of 12/31/2025; 30-month threshold=material gate long-term survival benchmark. Days in vivo (all arches): 622.8±373.7 (range 27-1036) (right). LDP: Lucitone Digital Print; SRHI: SprintRay High Impact

**Figure 9:**
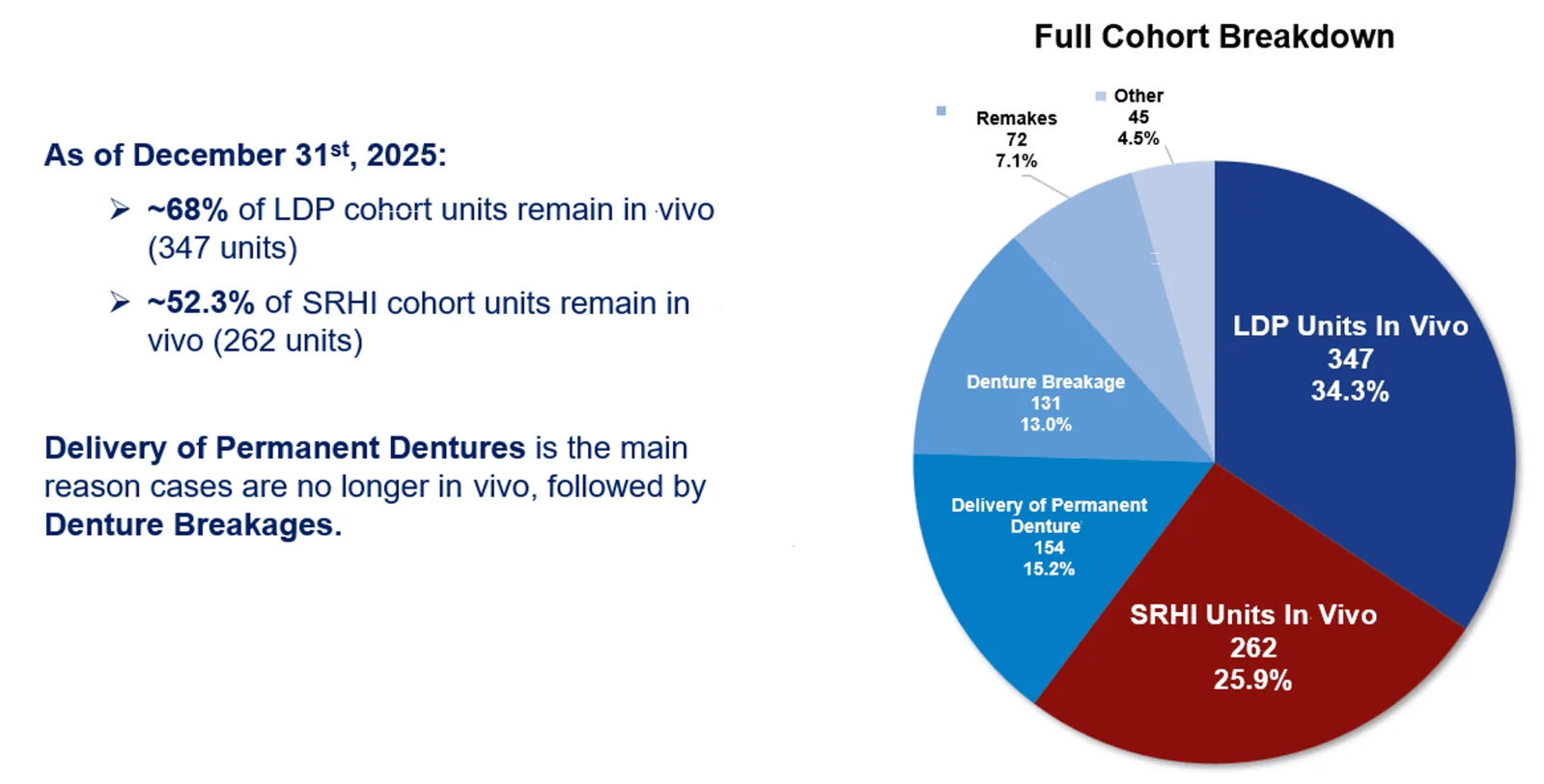
Material gate cohort: current state breakdown. LDP n=510 arches; SRHI n=501 arches; in vivo status as of 12/31/2025. Days in vivo: LDP 762±338; SRHI 622.8±373.7.

Sequential cohort analysis further demonstrated progressive increases in cumulative breakage rates from 2023 through 2025, while the majority of the cohort remained functionally intact at each interval, supporting overall material durability under real-world conditions [10-13] (Figure 6). Comparison of delivered versus failed units confirmed that both materials demonstrated similar overall fracture proportions, reinforcing that resin selection alone did not dictate clinical performance (Figure 7). Evaluation of manufacturing location and denture type demonstrated that fractures were distributed across both centralized and in-office workflows, with no single fabrication pathway exclusively associated with failure (Figure 8, left). Ordered time-to-failure analysis revealed clustering of fracture events within the early service period, followed by a plateau phase consistent with mechanical stabilization after initial functional adaptation (Figure 8, right). The majority of fractured prostheses were newly designed dentures, with fewer failures occurring in remake or reprint cases, indicating that initial design execution represents the highest-risk phase of the workflow (Figure 9).

**Figure 10:**
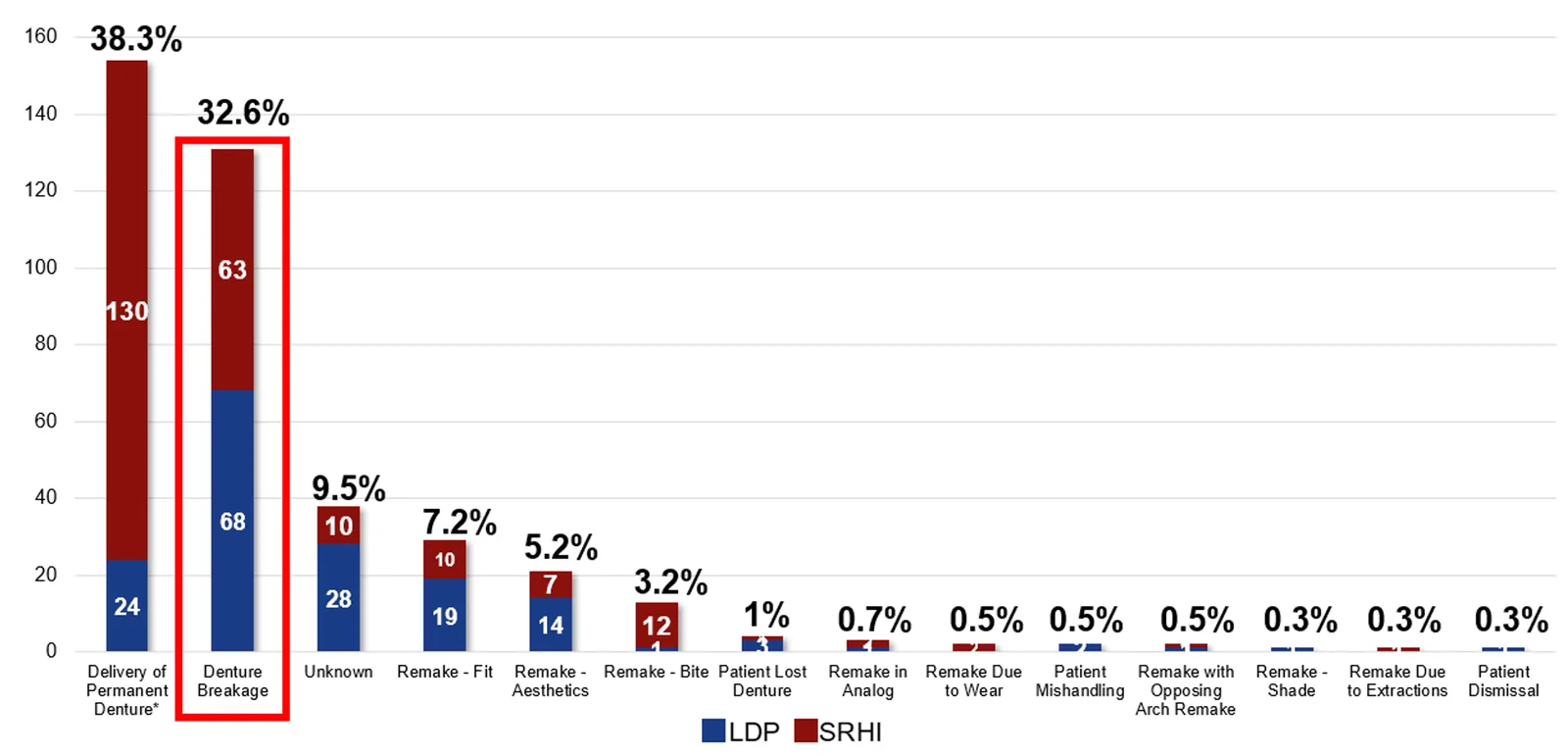
Reasons that cohort dentures are no longer in vivo. Percentages from total arches no longer in vivo across LDP and SRHI cohorts (n=326: LDP 140, SRHI 186). *Permanent dentures: a temporary denture later replaced by a permanent denture. LDP: Lucitone Digital Print; SRHI: SprintRay High Impact

**Figure 11:**
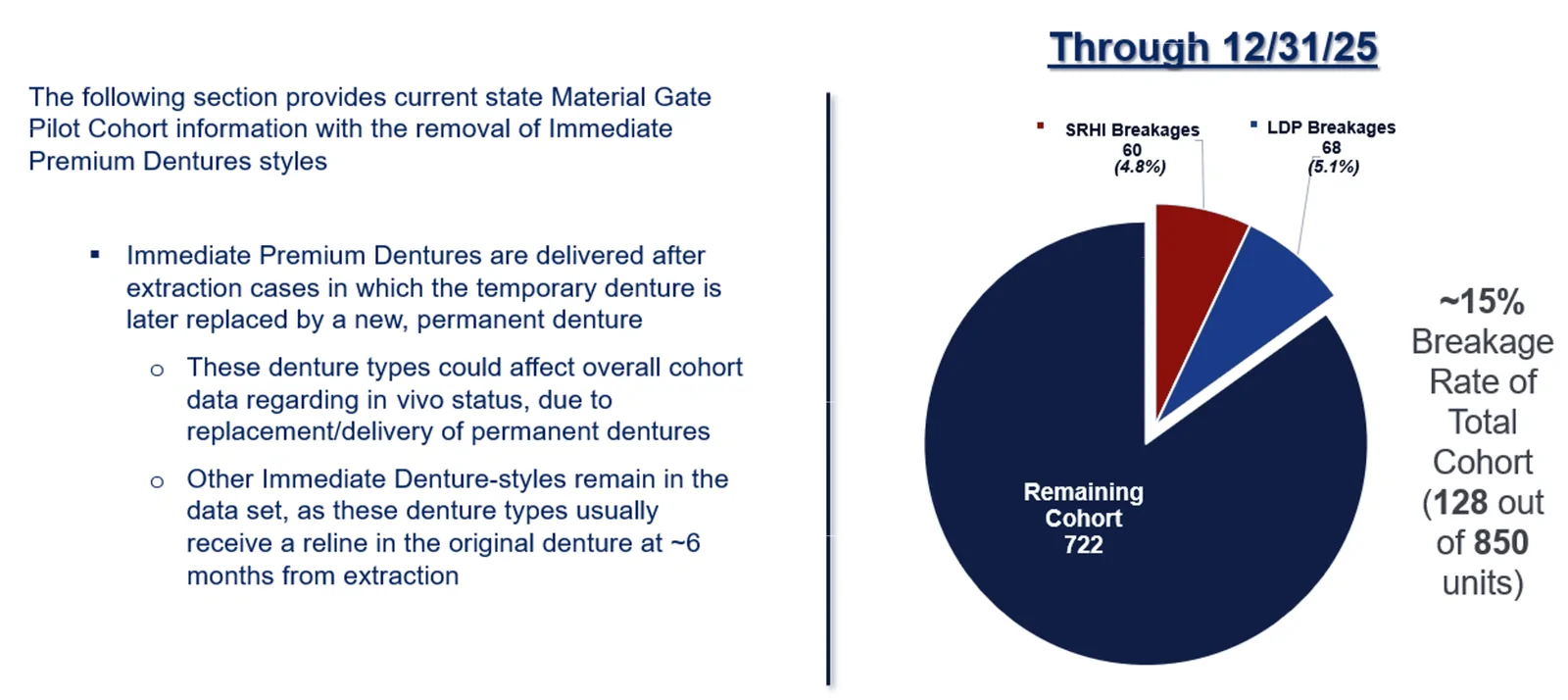
Material gate pilot data (no immediate premium dentures). LDP n=486 arches; SRHI n=364 arches. Days in vivo: LDP 781±329 (range 9-1072); SRHI 700.4±354.7 (range 27-1036). LDP: Lucitone Digital Print; SRHI: SprintRay High Impact

**Figure 12:**
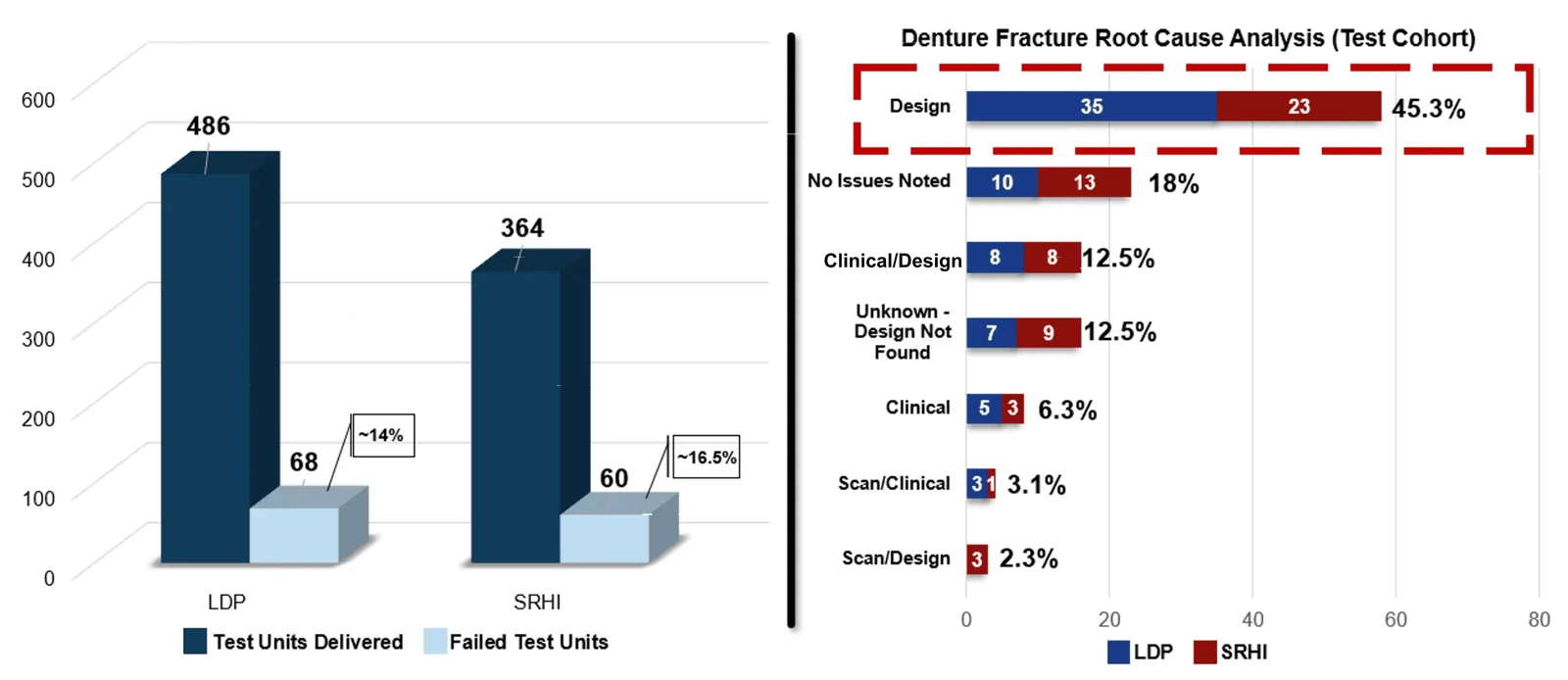
Cohort denture fractures and breakage reason (no immediate premium dentures). Broken arches: LDP n=68/486 (14.0%), SRHI n=60/364 (16.5%) (total n=128). Time-to-failure mean±SD: LDP 456.9±292.8 days (n=68); SRHI 368.1±269.3 days (n=60); combined 415.3±285.5 days. LDP: Lucitone Digital Print; SRHI: SprintRay High Impact

**Figure 13:**
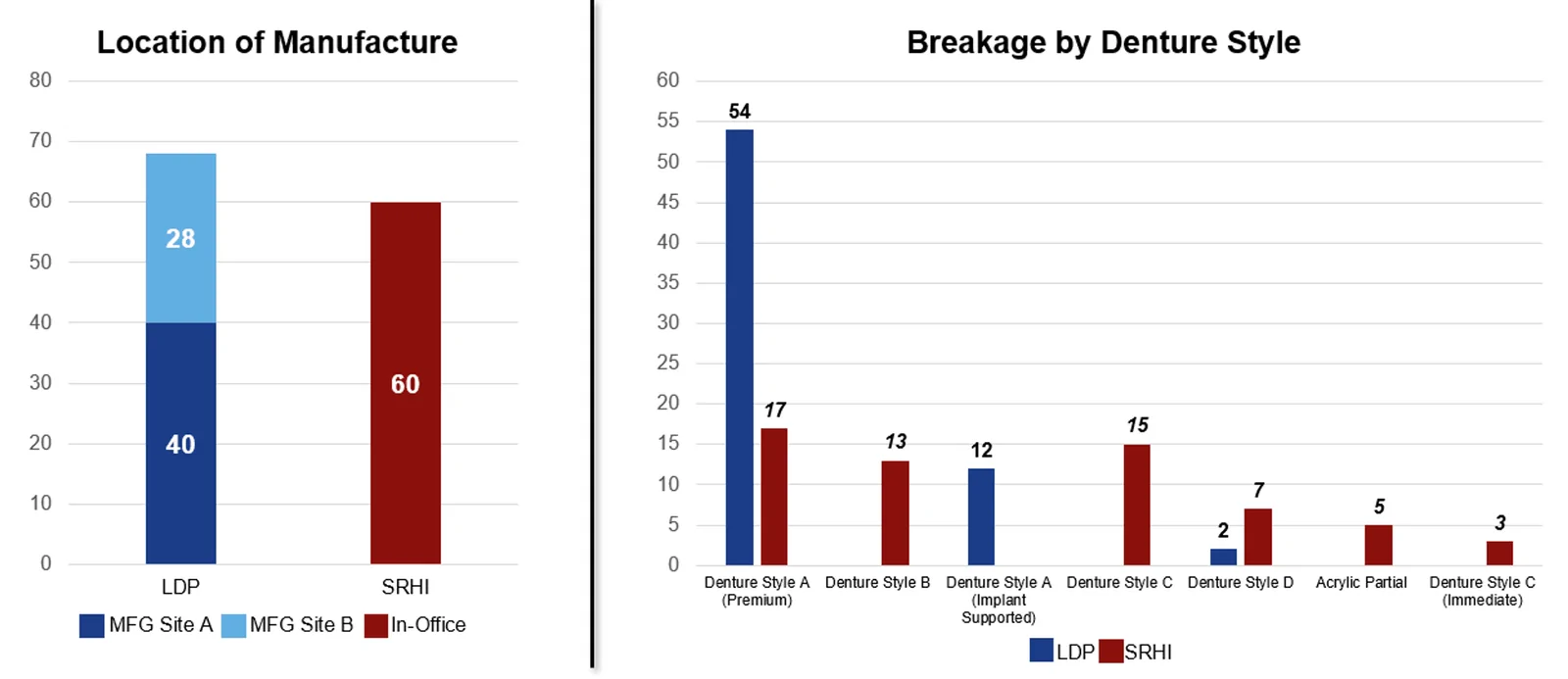
Cohort denture breakage analysis (no immediate premium dentures). LDP n=486 arches; SRHI n=364 arches. Time-to-failure mean±SD: LDP 456.9±292.8 days (n=68); SRHI 368.1±269.3 days (n=60); combined 415.3±285.5 days. LDP: Lucitone Digital Print; SRHI: SprintRay High Impact

Analysis of high-frequency fracture locations demonstrated clustering within specific clinical sites, suggesting that localized workflow variables or operator technique contributed to increased failure incidence (Figure 10). Within these outlier locations, fracture etiology remained dominated by design-related and combined clinical/design factors, further supporting the role of workflow execution in failure patterns (Figure 11). Longitudinal analysis of in vivo service demonstrated sustained performance for LDP, with approximately 68% of units remaining functional beyond 30 months (Figure 12). In contrast, SRHI units demonstrated a lower proportion of long-term in vivo retention, with approximately 52% remaining functional beyond 30 months (Figure 13).

**Figure 14:**
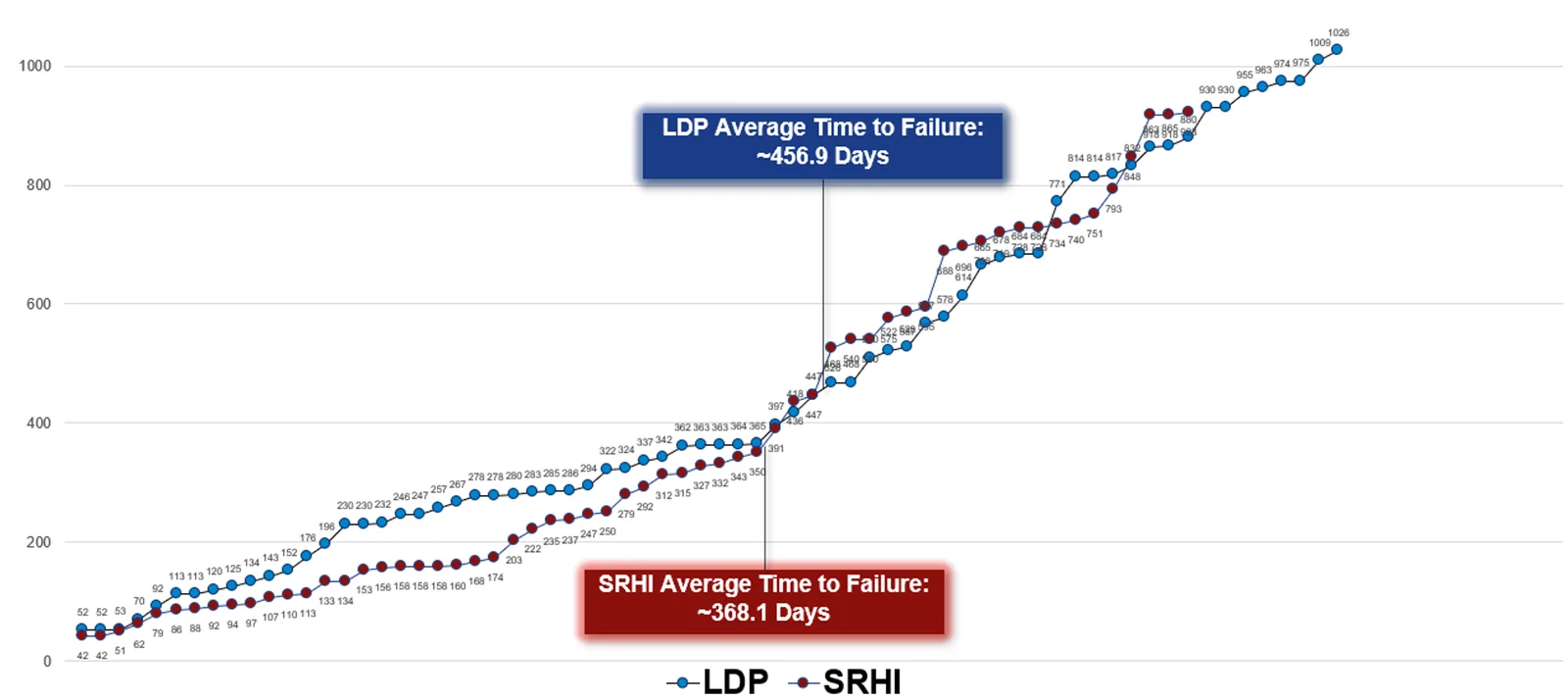
Time to failure: cohort denture breakages (no immediate premium dentures). Time to failure of broken arches with end-of-service date (n=128). Mean±SD: LDP 456.9±292.8 days (n=68); SRHI 368.1±269.3 days (n=60); combined 415.3±285.5 days.

**Figure 15:**
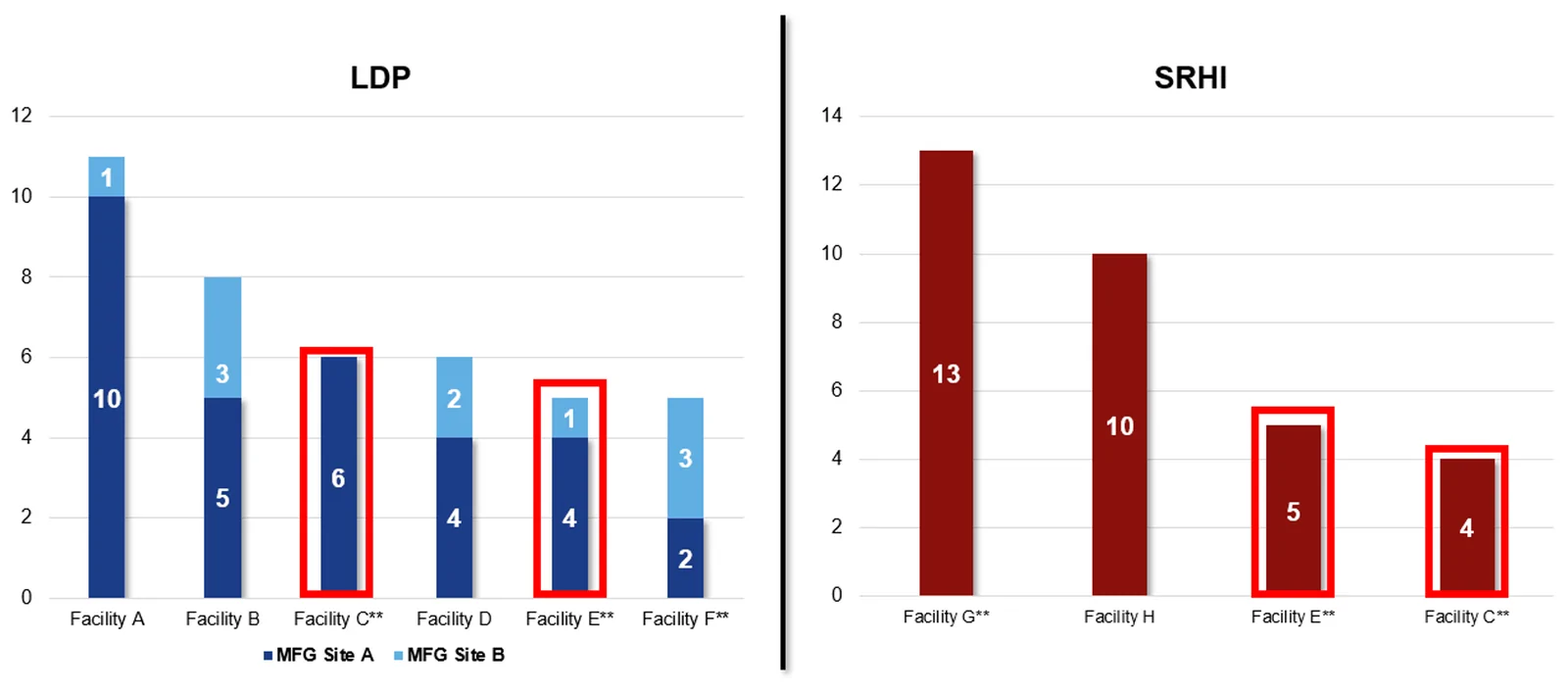
Cohort denture breakages: top outlier offices (no immediate premium dentures). **Offices with in-office designers at fabrication. Offices ranked by broken-arch count (excl. immediate premium denture styles). Total broken arches: n=128 (LDP 68, SRHI 60). Time-to-failure mean±SD: LDP 456.9±292.8 days (n=68); SRHI 368.1±269.3 days (n=60); combined 415.3±285.5 days. LDP: Lucitone Digital Print; SRHI: SprintRay High Impact; MFG: manufacturing

**Figure 16:**
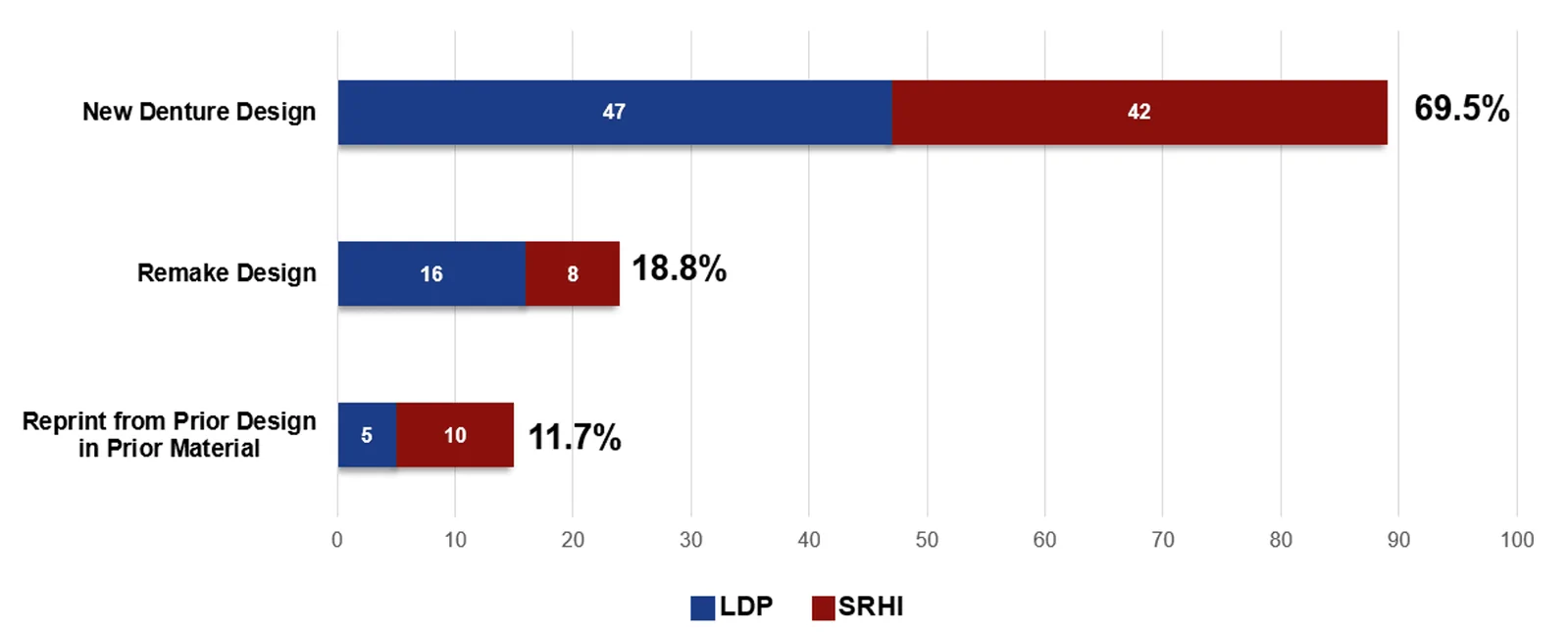
Cohort group: denture type prior to breakage (no immediate premium dentures). Denture style at fabrication tabulated across broken arches (excl. immediate premium denture styles). Total broken arches: n=128 (LDP 68, SRHI 60). Combined time-to-failure mean±SD: LDP 456.9±292.8 days (n=68); SRHI 368.1±269.3 days (n=60); combined 415.3±285.5 days. LDP: Lucitone Digital Print; SRHI: SprintRay High Impact

**Figure 17:**
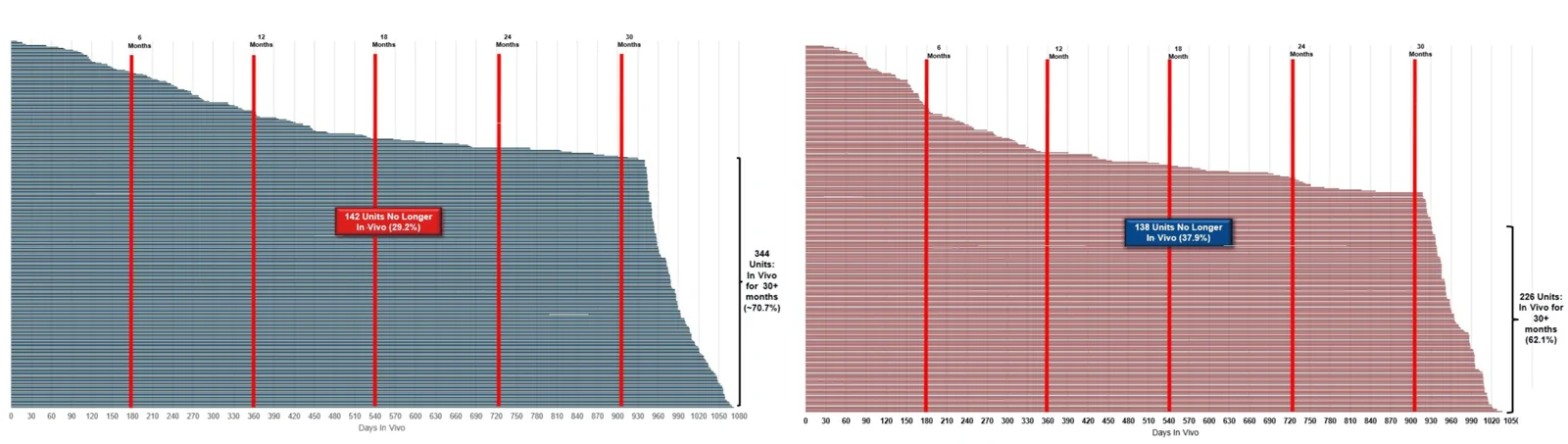
LDP material (left) and SRHI material (right) gate cohort time in vivo (no immediate premium dentures). LDP cohort excl. immediate premium denture styles: n=486 arches; in vivo status as of 12/31/2025. Days in vivo: 781±329 (range 9-1072) (left). SRHI cohort excl. immediate premium denture styles: n=364 arches; in vivo status as of 12/31/2025. Days in vivo: 700.4±354.7 (range 27-1036) (right). LDP: Lucitone Digital Print; SRHI: SprintRay High Impact

Cohort status analysis confirmed that denture breakage represented a significant but secondary cause of discontinuation, with delivery of definitive prostheses accounting for the largest proportion of units no longer in service (Figure 14). Detailed analysis of discontinuation pathways demonstrated that denture fracture was the second most common cause of removal, accounting for approximately 45.7% of cases following definitive prosthesis delivery (Figure 15). Evaluation of the pilot cohort excluding immediate premium dentures demonstrated similar fracture distribution patterns, confirming consistency of findings across cohort subsets (Figure 16). Root cause distribution within the pilot cohort mirrored the primary dataset, with design factors again representing the dominant contributor to fracture events (Figure 17, left). Manufacturing location analysis within the pilot dataset showed comparable distribution patterns, indicating that fabrication pathway alone did not predict failure (Figure 17, right).

**Figure 18:**
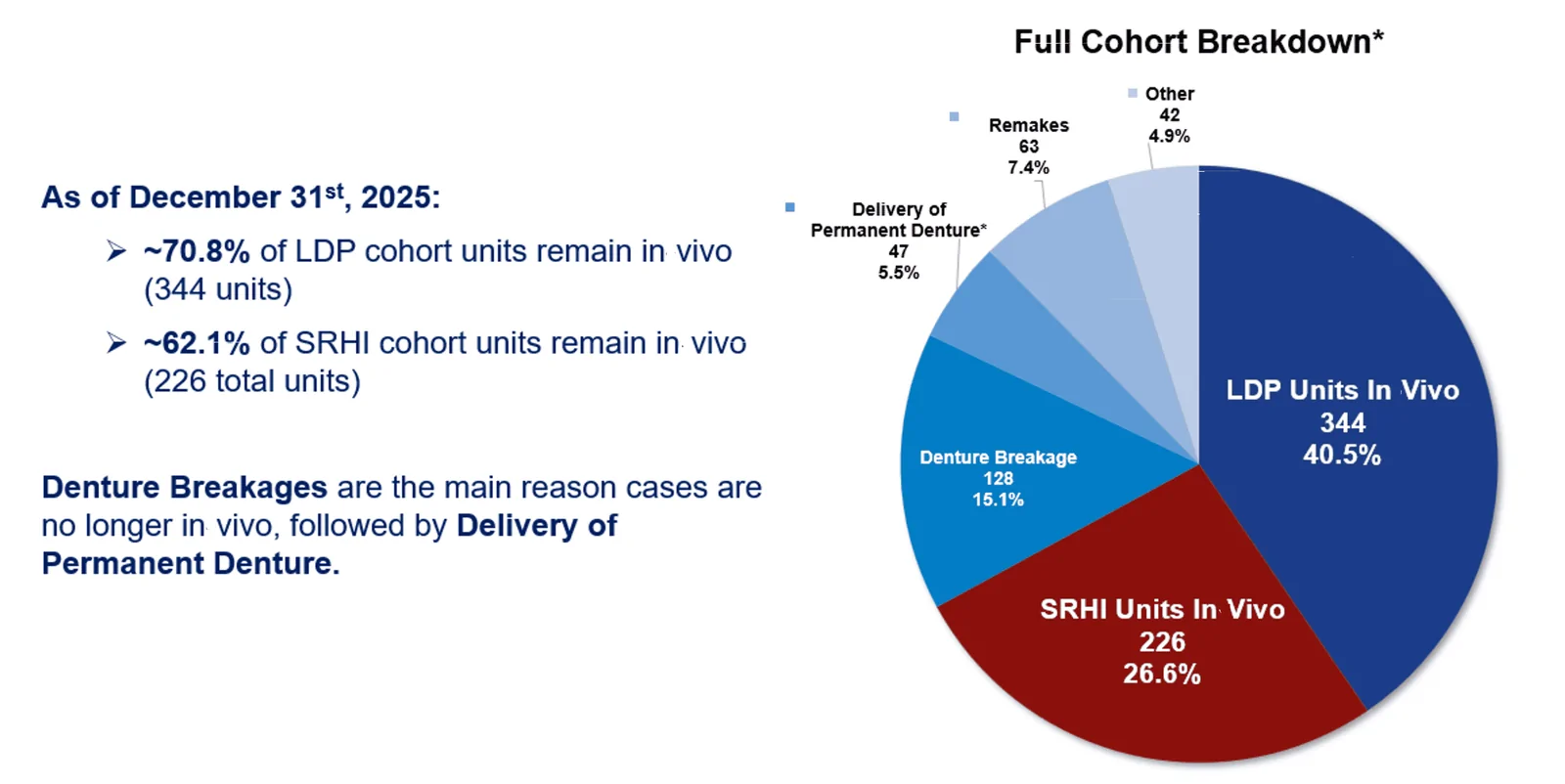
Material gate cohort: current state breakdown (no immediate premium dentures). *Excludes immediate premium denture styles. LDP n=486 arches; SRHI n=364 arches. In vivo status as of 12/31/2025. Days in vivo: LDP 781±329 (range 9-1072); SRHI 700.4±354.7 (range 27-1036). LDP: Lucitone Digital Print; SRHI: SprintRay High Impact

**Figure 19:**
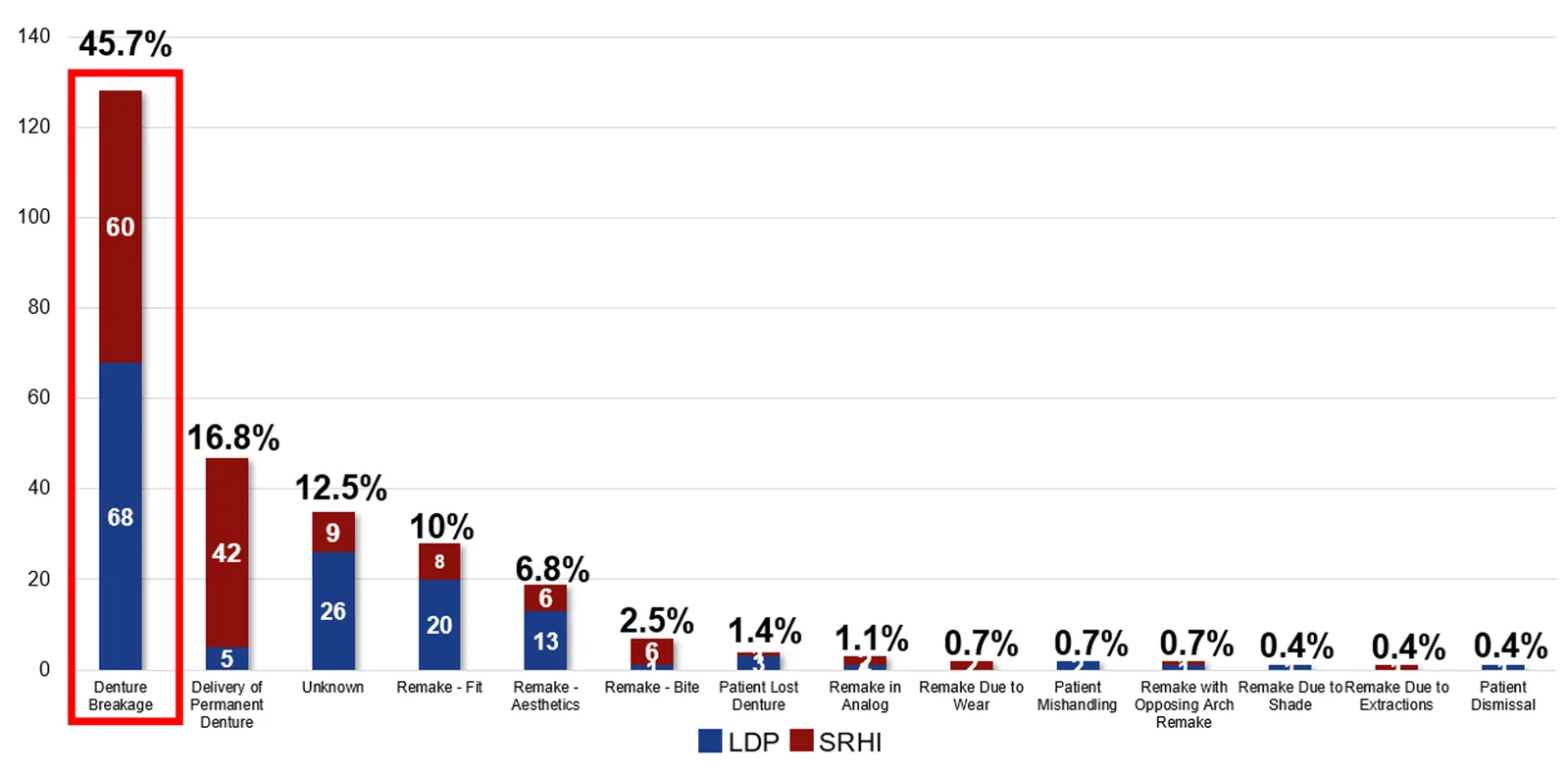
Reasons that cohort dentures are no longer in vivo (no immediate premium dentures). Percentages from total arches no longer in vivo (excl. immediate premium denture styles); n=280 arches (LDP 142, SRHI 138). LDP: Lucitone Digital Print; SRHI: SprintRay High Impact

Temporal failure trends in the pilot cohort demonstrated early clustering similar to the full dataset, reinforcing the importance of initial prosthesis adaptation (Figure 18). Final outlier analysis confirmed that fracture clustering was associated with specific clinical locations rather than material type, further supporting the conclusion that workflow execution, prosthesis design, and clinical technique are the principal determinants of clinical outcome (Figure 19). Patient and denture demographics of the two groups (LDP vs SRHI) in the full cohort are illustrated in Table 3.

**Table 3:** Patient and denture demographics: LDP vs SRHI full cohorts. Categorical data presented as N (%) and continuous data as mean±SD with range. LDP: Lucitone Digital Print; SRHI: SprintRay High Impact

Characteristic	LDP (patient (N=338); arch (N=510))	SRHI (patient (N=339); arch (N=501))	Category
Female patients	173 (51.2%)	184 (54.3%)	Gender
Male patients	148 (43.8%)	142 (41.9%)	Gender
Unspecified patients	17 (5.0%)	13 (3.8%)	Gender
Single arches	250 (49.0%)	249 (49.7%)	Arch
Dual arches	260 (51.0%)	252 (50.3%)	Arch
Arches in vivo: yes	347 (68.0%)	262 (52.3%)	Status
Arches in vivo: no	163 (32.0%)	239 (47.7%)	Status
Arch days in vivo (mean±SD)	762.3±337.5	622.8±373.7	Duration
Arch days in vivo (range)	9-1072	27-1036	Duration

## Discussion

This multicenter cohort demonstrates that contemporary high-impact 3D-printed denture resins perform successfully under routine clinical conditions. The most important finding is that functional retention differed more substantially than fracture-free survival. This distinction is clinically important because functional retention is influenced by planned definitive prosthesis delivery, remake decisions, clinical workflow, and practice preferences, whereas fracture-free survival more directly reflects structural material performance. Although the SRHI cohort demonstrated earlier functional discontinuation, fracture-free survival analysis showed only a modest material-associated hazard, suggesting that resin selection should not be interpreted independently of denture indication, fabrication workflow, and prosthesis design. The SRHI cohort also contained a higher proportion of immediate dentures and chairside workflows, both of which may have contributed to early discontinuation independent of intrinsic material performance.

Although these findings suggest that workflow execution and prosthesis design may contribute substantially to long-term clinical performance, these associations should be interpreted cautiously. Workflow-related variables, including fabrication pathway and denture indication, were not prospectively controlled or statistically adjusted because of the observational multicenter study design and incomplete covariate data. Consequently, the observed relationships represent associations rather than evidence of causation and should be considered hypothesis-generating. Future prospective studies using standardized clinical protocols and multivariable analyses are needed to better define the independent contribution of workflow factors to clinical outcomes.

Importantly, these findings demonstrate that high-impact printed denture materials perform favorably under real-world clinical conditions and compare well with competitive 3D-printed denture materials currently available. The favorable clinical performance observed with this generation of high-impact resin supports the continued evolution of additive denture materials. Since the completion of this clinical dataset, SprintRay has introduced the Apex family of denture materials, which retains the proven high-impact resin chemistry while incorporating enhanced translucency, improved life-like esthetics, and superior mechanical properties. Although long-term clinical studies are still needed, these improvements suggest that Apex may provide clinical performance that equals or exceeds that observed in the present investigation.

The data further reinforce that 3D-printed dentures should be evaluated as engineered prostheses rather than simply as resin specimens [21-25]. Inadequate design thickness, suboptimal occlusal schemes, insufficient reinforcement of high-stress zones, and inaccurate tissue capture may create stress concentrations that exceed the material tolerance of otherwise acceptable resins [11-13]. The clinical implication is that printed dentures require the same prosthodontic principles that govern conventional dentures, but those principles must be deliberately translated into the digital design environment [15-19].

Biomechanical considerations unique to additive denture fabrication

Additive manufacturing introduces anisotropic material behavior [10-12]. Digital dentures also lack the compressive adaptation associated with conventional flasking and polymerization. As a result, tissue adaptation depends primarily on the accuracy of digital impression capture, CAD, print precision, and controlled post-processing [26,27]. Localized instability may generate flexure during mastication and contribute to fracture, especially in mandibular dentures and immediate dentures undergoing continued ridge remodeling. These findings emphasize that successful printed dentures require sound prosthodontic principles translated into the digital environment.

Clinical integration of contemporary digital workflows

One of the most important developments since the introduction of printed dentures has been the maturation of the complete digital workflow. Earlier digital denture fabrication required considerable laboratory expertise and multiple manual processing steps. Contemporary SprintRay workflows have been simplified and streamlined to the point that trained dental staff can efficiently perform much of the fabrication process within the dental office.

Advances in both hardware and software have substantially reduced production complexity while improving consistency. Systems such as the SprintRay Pro2 equipped with DuoKit technology have optimized manufacturing efficiency and now allow true same-day denture fabrication for many clinical situations. Automated workflows encompassing digital scanning, CAD, printing, washing, and post-curing have reduced operator dependence while improving reproducibility and clinical consistency [28,29].

The economic advantages of current SprintRay workflows are equally important. The SRHI denture resin is approximately 15% less expensive than the LDP resin, based on the manufacturer's published list pricing at the time of manuscript preparation, while providing improved translucency, enhanced life-like esthetics, and excellent clinical performance in this multicenter cohort. Combined with simplified manufacturing, reduced labor requirements, and rapid chairside or laboratory fabrication, these workflow improvements substantially decrease fabrication time and overall production costs compared with conventional processing or subtractive milling. The simplified workflow lowers the technical barrier for clinicians wishing to incorporate in-office denture fabrication, allowing more practices to provide removable prosthodontic treatment efficiently. These efficiencies have the potential to expand patient access to high-quality dentures while reducing treatment time and overall treatment costs.

The clinician-laboratory relationship likewise continues to evolve toward collaborative digital treatment planning. Design files can be evaluated before manufacturing, archived indefinitely, reproduced when replacement prostheses are required, and modified when clinical experience identifies recurring design deficiencies. This ability to preserve and continuously improve validated prosthesis designs represents one of the greatest long-term advantages of digital removable prosthodontics [29].

Limitations

This study was conducted as a real-world multicenter cohort rather than a randomized controlled trial. Differences in denture type, indication, practice workflow, fabrication pathway, and patient characteristics may have influenced survival outcomes. The SRHI cohort included a greater proportion of immediate dentures and chairside workflows, both of which may have increased discontinuation unrelated to intrinsic resin performance. Some prostheses were removed from service because of planned definitive prosthesis delivery, remake, or clinical decision-making rather than fracture. Accordingly, functional retention and fracture-free survival were analyzed separately.

The comparison of material costs reflects the manufacturer's published list pricing available at the time of manuscript preparation and does not account for regional pricing differences, distributor discounts, or future pricing changes.

Future prospective studies incorporating standardized follow-up intervals, patient-level event adjudication, and evaluation of newer-generation materials such as Apex will further clarify the relative contributions of material properties and workflow variables.

In addition, follow-up intervals were not standardized across participating centers, reflecting routine clinical practice rather than a protocol-driven evaluation. Inter-examiner calibration was not formally performed, and complete patient-, prosthesis-, and site-level covariate data were not consistently available for all participating centers. Consequently, multivariable adjustment for potential confounding variables could not be performed, and residual confounding cannot be excluded. These factors may limit internal validity and reproducibility but are inherent to the real-world multicenter observational design of the present investigation.

We note that samples in this study originated from a decentralized network of clinical sites, encompassing variation in denture type, immediate denture use, in-office fabrication protocols, and post-curing procedures. While the current Instructions for Use (IFU) and standard operating procedures (SOPs) for these materials and workflows are clearly defined and designed to be user-friendly, a decentralized fabrication environment inherently carries less centralized oversight compared to a controlled laboratory or single-site setting. As a result, there is a greater potential for site-to-site variability in strict adherence to IFU/SOP parameters, such as post-curing time, light exposure, or handling technique, even when protocols themselves are well-established and accessible.

Clinical design recommendations

**Figure 20:**
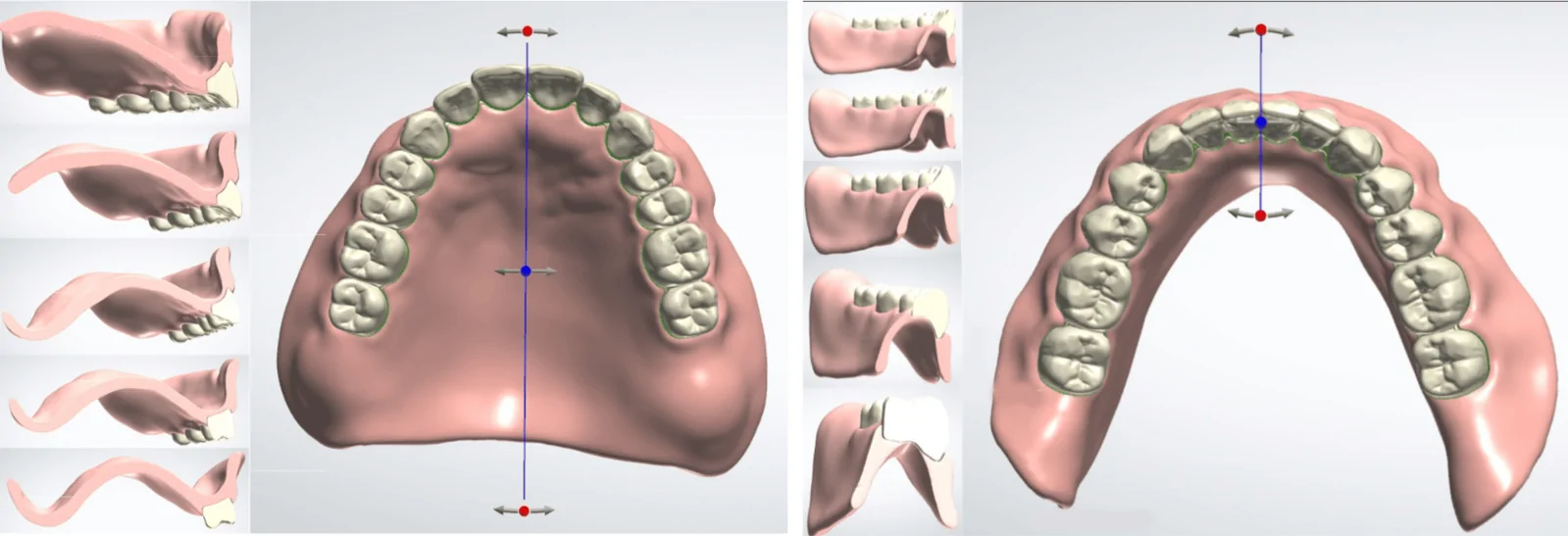
Virtual design of the maxillary (left) and mandibular (right) denture demonstrating ideal thickness of the printed denture base at the various teeth on the denture. The figure was created manually using the 3Shape software (3Shape, New Providence, NJ, USA).

**Figure 21:**
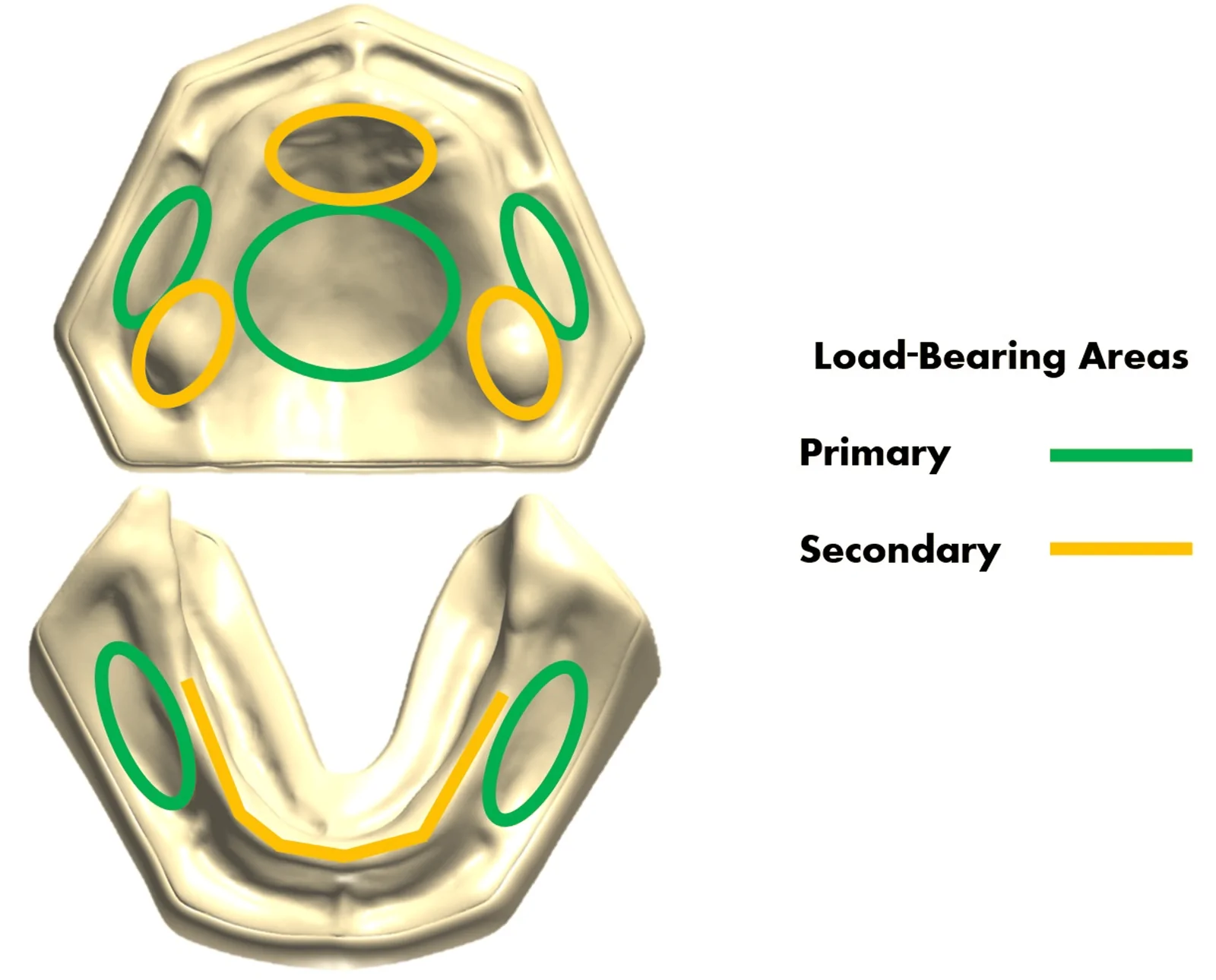
Load-bearing areas on the edentulous arches that affect denture survival during function. The figure was created manually using the 3Shape software (3Shape, New Providence, NJ, USA).

Minimum denture base thickness should be maintained at 2.5-3.5 mm in stress-bearing regions, especially across the mandibular midline (Figure 20 and Figure 21). Thin mandibular ridges and parafunctional patients may benefit from reinforcement strategies including fiber incorporation or metal framework support. Occlusal design should incorporate digitally simulated bilateral balance while minimizing cantilevered force vectors to reduce tensile stress within the denture base. Digital verification protocols should confirm accurate border extension, intaglio adaptation, tissue capture, and occlusal simulation before fabrication.

Reprints may be appropriate when fracture results from isolated processing defects and the original structural design remains sound. In contrast, complete remakes are indicated when inadequate thickness, occlusal imbalance, or design deficiencies are identified as the primary cause of failure, allowing the correction of adaptation and biomechanical support.

Risk factors for early printed denture failure

Fracture clustering during the first year suggests that biomechanical adaptation and digital workflow execution strongly influence long-term durability. Immediate dentures demonstrated one of the highest early failure risks because rapid post-extraction ridge remodeling alters intaglio adaptation and occlusal relationships, producing uneven load distribution and increased flexural strain across the mandibular midline.

Thin mandibular denture bases remain a primary structural weakness because mandibular flexure concentrates tensile stress within reduced-thickness regions, particularly at the lingual flange and midline [30-32]. Opposition by natural dentition further increases fracture risk because of higher occlusal forces and localized contact loading. Bruxism and clenching accelerate cyclic fatigue failure, making careful occlusal adjustment and reinforcement increasingly important in these patients.

Digital workflow inaccuracies also contribute significantly to early failure. Poor scan fidelity, incomplete tissue capture, inaccurate border registration, and occlusal imbalance may create instability and concentrated stress zones within the prosthesis. Additional contributing factors include inadequate post-curing, unfavorable print orientation, and limited experience with digital design software. Collectively, these findings demonstrate that biologic remodeling, biomechanical design, and digital workflow execution exert greater influence on long-term clinical durability than resin selection alone.

Future directions

Future investigations should isolate individual workflow variables to quantify their relative contributions to prosthesis failure. Controlled studies evaluating print orientation, support strategy, layer thickness, post-curing protocol, and CAD reinforcement design would provide valuable guidance for optimizing printed denture performance. Longer-term longitudinal studies extending beyond 3-5 years are needed to determine whether newer high-impact denture materials, including Apex, demonstrate fatigue resistance comparable with or superior to conventional PMMA over extended service periods. The integration of artificial intelligence into CAD may further improve clinical outcomes by identifying high-risk stress regions before fabrication.

## Conclusions

High-impact 3D-printed denture resins demonstrated excellent clinical performance in this large multicenter cohort and compared favorably with competitive 3D-printed denture materials currently available. Functional retention differed between LDP and SRHI, but fracture-free survival demonstrated that prosthesis indication, digital workflow, prosthesis design, and manufacturing consistency exerted a greater influence on long-term clinical success than material selection alone.

The findings also demonstrate the remarkable maturation of digital denture technology. Contemporary SprintRay workflows have simplified fabrication to the point that trained dental personnel can efficiently produce high-quality dentures within the dental office. Hardware innovations such as the SprintRay Pro2 with DuoKit technology support true same-day denture fabrication, while advances in printable materials continue to improve both clinical performance and esthetics.

The SRHI resin evaluated in this study provides enhanced translucency and improved life-like esthetics while reducing material costs by approximately 15% compared with LDP resin (based on the manufacturer's published list pricing at the time of manuscript preparation). The subsequent introduction of the Apex family of denture materials, incorporating the same proven high-impact resin chemistry together with enhanced esthetics and improved mechanical properties, represents the next evolution of digital denture materials and warrants prospective clinical investigation to determine whether these improvements translate into superior long-term clinical outcomes.

Both materials demonstrated similar fracture performance (LDP 13.3% vs SRHI 12.6%; HR: 1.18; 95% CI: 0.84-1.67; P=.341, not statistically significant), while overall functional retention did differ meaningfully between groups. This retention difference is more likely attributable to workflow-related factors, including denture indication (e.g., immediate denture protocols), planned transition to definitive prostheses, and variability in clinical protocol, rather than an inherent difference in material performance. Thus, this indicates that structural/mechanical failure was not a meaningful driver of the difference observed in overall functional retention; rather, design quality and workflow execution (not resin composition) were the primary determinants of fracture risk. The retention findings should therefore not be interpreted as reflecting a meaningful difference in the mechanical performance of the two materials themselves.

Overall, the long-term success of digitally fabricated dentures depends on accurate digital data acquisition, thoughtful CAD, controlled manufacturing, and standardized post-processing. When these variables are optimized, contemporary high-impact 3D-printed dentures provide a clinically predictable, economically efficient, esthetically pleasing, and increasingly accessible treatment option for removable prosthodontics.
